# Evaluation of Cytomegalovirus Infection after Six Months of Liver Transplantation in Children in Shiraz, Southern Iran

**Published:** 2011-02-01

**Authors:** N. Honar, M. H. Imanieh, S. M. Dehghani, M. Haghighat, B. Geramizadeh, R. Yaghobi, A. Alborzi, M. Ziaeian, K. Kazemi, S. Nikeghbalian, A. Bahador, H. Salahi, S. A. Malek Hosseini

**Affiliations:** 1 *Gastroenterohepatology Research Center, *; 2 *Shiraz Transplant Research Center, *; 3 *Clinical Microbiology Research Center, Nemazee Hospital, Shiraz University of Medical Sciences, Shiraz, Iran*

**Keywords:** Children, Liver transplantation, Late-onset cytomegalovirus infection

## Abstract

Background: Liver transplantation (LT) is a life-saving treatment for end-stage liver diseases (ESLD). Cytomegalovirus (CMV) infection is one of the important causes of morbidity after LT.

Objective: To evaluate the incidence of late-onset (after 6 months of LT) CMV infection in pediatric recipients.

Methods: A retrospective analysis was conducted to evaluate 50 pediatric patients who underwent LT for 8 years at the LT Unit of Nemazee Hospital affiliated to Shiraz University of Medical Sciences, Shiraz, Iran. We retrospectively investigated episodes of CMV infection after 6 months of LT proven by CMV antigenemia test.

Results: Three recipients (6%) developed late-onset CMV infection. These patients finally responded to ganciclovir.

Conclusion: CMV infection is one of the most common post-LT viral infections that usually occurs in the first six months of LT. Our study shows that the incidence of late-onset CMV infection is relatively low, but it still remains a significant problem. Therefore, monitoring and management is crucial for improving the survival of children.

## INTRODUCTION

Liver transplantation (LT) is a life-saving treatment for many forms of chronic and end-stage liver diseases (ESLDs) in pediatric age groups [1, 2].

Survival of pediatric patients has significantly improved during the last decade, which is mostly due to improvement in the diagnosis, surgical techniques, immunosuppressive medications and antimicrobial agents [3].

Infections and their complications remain the leading cause of morbidity and mortality after LT. Risk factors such as surgical procedure and the use of immunosuppressive medications after LT, lead to higher incidence of infection in children [4]. Careful monitoring and management of these infections can decrease morbidity and mortality and improve the outcome of these patients [5, 6]. 

Cytomegalovirus (CMV) infection is an important post-transplant opportunistic infection in children that can increase morbidity and mortality after LT [7]. Although several studies have evaluated early-onset CMV infection (during first six months of transplantation), few studies have been conducted on late-onset (after six months of LT) infection. 

The objective of this study was to determine the incidence of late-onset CMV infection in pediatric liver recipients.

## PATIENTS AND METHODS

In a cross-sectional study, we examined 50 pediatric LT recipients from the LT Center of Shiraz University of Medical Sciences (SUMS), Shiraz, Iran. The patients aged between 1 and 18 years.

All of the children were followed in LT clinic, Nemazee Hospital affiliated to Shiraz University of Medical Sciences by LT surgeons and pediatric hepatologists group between 6 and 96 months. If they had any signs or symptoms of the infection including fever, diarrhea, or leukopenia, they were evaluated by CMV antigenemia test and if needed, admitted to hospital for management. Immunofluorescence techniques have been used for CMV antigenemia test. 

According to clinical manifestations and the status of CMV antigenemia, the patients were categorized into three groups:

Patients with CMV antigenemia <7 who did not need any treatment for CMV infection;

Patients with CMV antigenemia between 7 and 12 who were followed by another CMV antigenemia and if the second test increased to >12 antiviral therapy was started for them; and

Patients with CMV antigenemia >12 who were admitted for management.

We treated our patients with intravenous ganciclovir for at least three weeks and then continued treatment with oral valganciclovir until CMV antigenemia become negative.

## RESULTS

We studied 50 children—32 boys (64%) and 18 girls (36%). They had a mean±SD age of 10.6±4.6 (range: 1–18) years. Causes of LT in these children are shown in Table 1

**Table 1:** Causes of liver transplantation in the studied patients

**Cause**	**n (%)**
Extra-hepatic biliary atresia (EHBA)	13 (26)
Wilson’s disease	8 (16)
Tyrosinemia	7 (14)
Progressive familial intra-hepatic cholestasis (PFIC)	6 (12)
Autoimmune cirrhosis	5 (10)
Cryptogenic cirrhosis	1 (2)
Criggler Najjar syndrome	1 (2)
Fulminant hepatitis	1 (2)
Congenital hepatic fibrosis	1 (2)
Primary sclerosing cholangitis	1 (2)
Neonatal hepatitis	1 (2)
Hypercholesterolemia	1 (2)
Other	4 (8)
Total	50 (100)

Out of the 50 studied children, three (6%: 95% CI: 0%–13%) developed symptomatic CMV infection after six months of LT. These children received tacrolimus to attain a therapeutic blood level. 

Two patients developed fever and anorexia and admitted to pediatric hepatology ward for further evaluation. In these children, CMV antigenemia test were positive. Intravenous ganciclovir (10 mg/kg/day for three weeks) was started for the patients; they got better.

The third case was a child with fever and protracted watery diarrhea. After evaluation and work up, CMV antigenemia test was positive. The patient responded to intravenous ganciclovir (same dosage and duration) and got better.

## DISCUSSION

Infectious complication after pediatric LT remains one of the most common causes of morbidity and mortality [8]. A recent study on 2291 children revealed that severe infectious complications occur in 52% of patients within 15 months of LT [6]. Many studies showed that most infections occur within six months of LT [9, 10]. Infections become relatively uncommon after six months of LT and these infectious complications are primarily associated with chronic rejection, re-transplantation, or large doses of immunosuppressive therapy [5].

The incidence of viral infections after LT is higher in children and have poorer outcome than adults; that is due to high incidence of primary infection, especially with CMV in younger seronegative recipients [7, 11]. CMV seems to be the main cause of viral infection [11]. Most CMV infections occur between three and eight weeks after transplantation [12]. The likelihood of CMV infection becomes particularly high after LT from a CMV-positive donor into a CMV-negative recipient. When a mismatch cannot be avoided, the recipient will give prophylactic treatment post-operatively. 

Over the last decade, a better understanding of risk factors and the use of prophylactic ganciclovir for the prevention of CMV have dramatically changed the rate of infection with this virus. However, several research studies showed that the leading cause of mortality in infected patients is due to viral etiology. For example, one study revealed that 18 episodes of CMV infections (42%) occurred in 42 pediatric patients [5]. The majority of infection episodes occurred between one and six months, and only one developed after six months of LT. 

Late-onset CMV infection, occurring after the discontinuation of prophylaxis, has emerged as a problem in recent years. It is thought that it is due to use of a potent antiviral agent for a long period, which inhibits the development of long-term protective immunity against CMV. In a study of 64 patients, the incidence of late-onset CMV infection was 12.5% with 4.7% disease [13]. In another study in USA, late-onset CMV infection was developed in 19 (7.3%) of 259 recipients and was independently associated with an increased risk of mortality during the first post-transplantation year [14].

We found that 6% of children developed CMV infection after six months of LT. In our center, regardless of the recipients and donors CMV status before LT and prophylactic ganciclovir therapy, the rate is almost the same as previous reports [13-15]. Although the incidence of CMV infection of LT recipients in our center is relatively low, late-onset CMV infection is still an important problem. Future strategies to combat late-onset CMV infection include prophylaxis with monitoring for early detection of asymptomatic CMV infection, pre-emptive therapy and adjustment for immunosuppression. Although, the new therapeutic procedures and the use of modern diagnostic methods have reduced the incidence of severe infections, CMV still remains a significant pathogen in LT. Therefore, appropriate attention should be paid to diagnose and treat the late-onset CMV infection. Late-onset CMV infection and the choice of optimal prevention strategy remain the main CMV-associated challenges of these years.
